# Template-Free Synthesis of g-C_3_N_4_ Nanoball/BiOCl Nanotube Heterojunction with Enhanced Photocatalytic Activity

**DOI:** 10.3390/nano12152569

**Published:** 2022-07-27

**Authors:** Longfei Wang, Zheyuan Fan, Xixi Cao, Panfeng Fan, Yu Xie, Qing Sun, Jinsheng Zhao

**Affiliations:** 1 College of Environment and Chemical Engineering, Nanchang Hangkong University, Nanchang 330063, China; clwlf12@163.com (L.W.); fzy18367301997@163.com (Z.F.); cxx18231314@163.com (X.C.); sunqing@nchu.edu.cn (Q.S.); 2 Shandong Key Laboratory of Chemical Energy Storage and Novel Cell Technology, College of Chemistry and Chemical Engineering, Liaocheng University, Liaocheng 252059, China

**Keywords:** template-free synthesis, one-step method, g-C_3_N_4_ nanoball, BiOCl nanotube, heterojunction, photocatalytic, solvothermal method

## Abstract

There are many reports on g-C_3_N_4_ nanosheet and BiOCl nanosheet, but few studies on other morphologies of g-C_3_N_4_ and BiOCl. Herein, a g-C_3_N_4_ nanoball/BiOCl nanotube heterojunction prepared by a simple one-step acetonitrile solvothermal method is reported. The XRD results prove that the g-C_3_N_4_/BiOCl composites can be prepared in one step. SEM results revealed that the g-C_3_N_4_ was spherical and the BiOCl was tubular. The HRTEM results indicate that g-C_3_N_4_ has an amorphous structure and that the (100) crystal plane of BiOCl borders the g-C_3_N_4_. Spherical g-C_3_N_4_ has a narrow band gap (approximately 1.94 eV), and the band gap of g-C_3_N_4_/BiOCl after modification was also narrow. When the BiOCl accounted for 30% of the g-C_3_N_4_/BiOCl by mass, the quasi-primary reaction rate constant of RhB degradation was 45 times that of g-C_3_N_4_. This successful preparation method for optimizing g-C_3_N_4_ involving simple one-step template-free synthesis may be adopted for the preparation of diverse-shapes and high-performance nanomaterials in the future.

## 1. Introduction

Owing to sustained global population growth, industrialization is accelerating, resulting in ongoing damage to natural and built environments. Environmental pollution, the energy crisis, global warming, and other issues are seriously affecting the quality of human life. An inexhaustible, clean, and pollution-free energy source, solar energy has become a topic of significant interest in recent years. Photocatalysis is a low-cost technology that converts solar energy into chemical energy and can be applied for the mitigation of environmental pollution and the production of green energy [1,2,3,4,5].

Graphite phase C_3_N_4_ (g-C_3_N_4_) is an excellent photocatalytic material with a large specific surface area and stable chemical properties. Its discovery has stimulated a wave of research involving modification, doping, and compounding [6,7,8,9,10,11,12]. To date, the most popular preparation method for g-C_3_N_4_ is high-temperature calcination. Most prepared g-C_3_N_4_ exhibits a two-dimensional (2D) flaky morphology, and reported band gaps are approximately 2.7 eV [13,14,15]. Preparation by low-temperature thermal polymerization is less often reported. Three-dimensional (3D) spherical g-C_3_N_4_ can be prepared by a low-temperature acetonitrile solvothermal method. It has a narrower band gap (approximately 2.0 eV) than the 2D flaky g-C_3_N_4_, which theoretically results in a wider range of visible light response [16]. Furthermore, the low temperature solvothermal method affords a higher yield. As a result, preparation by low-temperature thermal polymerization with spherical structures endow g-C_3_N_4_ with great potential in environmental engineering applications because of its easy recycling ability, optimized stability, and light utilization [17]. In view of the advantage that solvothermal method can prepare materials with diverse morphologies, we want to prepare g-C_3_N_4_ composites with new and special morphologies and explore their properties because the controlling morphology is considered as one of the preferred strategies to further improve the photocatalytic activity of the catalyst [18,19,20,21]. For example, the three-dimensional flower-like ZnO displayed four times higher activity than the one-dimensional scale-like ZnO for the degradation of methylene orange [22]. Moreover, by tuning the crystalline phase and morphologies of BiVO**_4_** crystal, Zhao et al. [23] found that the photocatalytic water oxidation activity for the well-defined BiVO_4_ crystal with monoclinic scheelite type can be 50 times of their regular tetragonal BiVO_4_ particles. Therefore, it is our desire to choose a suitable method to regulate the morphology of the catalyst to improve its photocatalytic activity. The one-pot method has a special reaction system and a complicated reaction process, which makes it possible to prepare materials with different morphologies compared with the traditional multistep method. Therefore, in this study, we used a simple one-pot method to prepare a g-C_3_N_4_/BiOCl composites heterojunction using Bi_2_O_3_ as the Bi source, cyanuric chloride and dicyandiamide as the raw materials for g-C_3_N_4_ synthesis, and acetonitrile as the solvent to improve the visible light response of globular g-C_3_N_4_ and its photocatalytic performance. The as-prepared g-C_3_N_4_/BiOCl composites not only exhibit a new morphology (the BiOCl is tubular) but also significantly enhanced photocatalytic activities for RhB degradation. Compared with the g-C_3_N_4_/BiOCl that has already been reported [24], we found that g-C_3_N_4_/BiOCl with special morphologies prepared by the one-pot method have a faster rate of degradation of RhB and great convenience, which indicates that our method may have broader environmental engineering application prospects.

## 2. Materials and Methods

### 2.1. Synthesis of Materials

#### 2.1.1. Raw Materials

Cyanuric chloride, dicyandiamide, bismuth oxide (Bi_2_O_3_), acetonitrile, absolute ethanol, deionized water, bismuth pentahydrate (Bi(NO_3_)_3_·5H_2_O), sodium chloride, rhodamine B (RhB), and sodium hydroxide (NaOH) were used.

#### 2.1.2. Synthesize of CB

**Figure 1:**
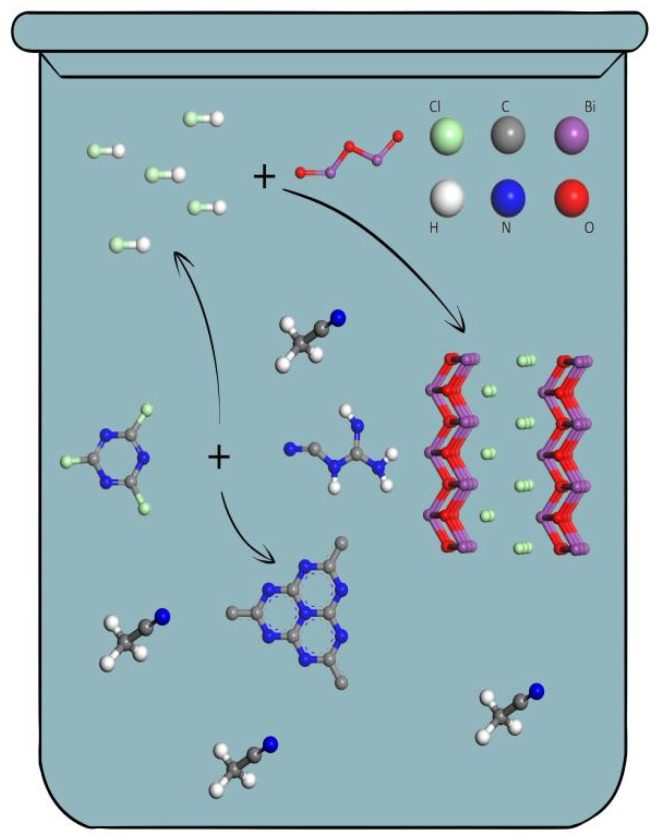
Reaction process of one-pot method.

g-C_3_N_4_/BiClO composites (denoted as CB) were prepared by a simple one-pot method (Figure 1). In a typical synthesis, 1.38 g cyanuric chloride and 0.42 g dicyandiamide were resolved in 30 mL of acetonitrile, and then added to a certain mass of bismuth oxide (Bi_2_O_3_), making the mass of BiClO account for 0%, 10%, 20%, 30%, and 40% of the mass of the composites. Then, the beaker was covered with plastic wrap and stirred at room temperature for 6 h. Finally, the mixture was transferred to a 50 mL high-pressure reactor, sealed in a stainless-steel hydrothermal reaction shell, and reacted at 180 °C for 24 h. The resulting precipitate was washed several times with acetonitrile and ethanol, and then dissolved in deionized water and adjusted to pH = 7 with sodium hydroxide. Then, the sample was placed in an oven at 80 °C to dry for 12 h and were abbreviated as CB-X (X = 0, 1, 2, 3, and 4). The CB-0 is pure g-C_3_N_4_.

#### 2.1.3. Synthesize of BiOCl

The BiOCl were synthesized by a simple solvothermal method. In a typical synthesis, Bi(NO_3_)_3_·5H_2_O (4 mmol), NaCl (4 mmol), and deionized water (35 mL) were mixed by vigorous stirring at ambient temperature for 6 h. Then, the above mixed solution was transferred into a 50 mL Teflon-lined stainless-steel autoclave and heated at 180 °C for 24 h. The samples were harvested by centrifugation, thoroughly washed with deionized water and ethanol several times, and finally dried at 80 °C for 12 h.

### 2.2. Characterization

The phase structure of the sample was characterized by XRD (D8ADVANCE-A25, Bruker, Billerica, MA, USA), and the morphology of the sample was characterized by field emission scanning electron microscopy (FEI Company, Hillsboro, OR, USA). The crystal plane spacing and elemental distribution of crystals were analyzed by Talos F200X FEI field emission transmission electron microscopy (ThermoFisher Scientific, Waltham, MA, USA). The XPS (Axis Ultra DLD KRATOS, Manchester, England, UK) was used to analyze the elemental composition and surface properties of materials. The UV-vis spectra (UV-2600, Shimadzu, Kyoto, Japan) was used to calculate the band gap of materials. Time-resolved fluorescence decay spectroscopy was measured using FLS1000 time correlated single-photon counting system (Edinburg Instruments, Edinburgh, Scotland, UK). The photoelectric properties of the samples were recorded in a standard three-electrode system on a CHI 660E electro-chemical workstation (Shanghai, China). A 300 W Xe arc lamp equipped with a 400 nm cut-off filter was used as the light source. Pt wire and Ag/AgCl electrode were employed as the counter electrode and reference electrode, respectively. The working electrode was obtained by deposition of as-prepared sample on 1 cm×1 cm FTO glass. All measurement was performed in 0.5 mol/L Na_2_SO_4_ aqueous electrolyte.

### 2.3. Photocatalytic Activity Experiment

The photocatalytic degradation experiment used a 300 W xenon lamp with a 420 nm cut-off filter (260 mW cm**^−^**^1^). Condensed water was passed through the outer layer of the beaker to ensure a constant temperature for the catalytic reaction. A certain weight of the catalyst (23 mg) was added to the beaker with 50 mL of organic pollutant solution (10 mg/L). Prior to illumination, the solution was magnetically stirred for 40 min in the dark to reach the adsorption equilibrium between the catalyst and organic pollutants. A 3 mL sample of the suspension was taken and centrifuged at given time intervals to measure the changes in the pollutant concentration during light irradiation. The concentration of the RhB and MO was measured with a UV-vis spectrophotometer at maximum absorption wavelength (λ = 550 and 463 nm, respectively). The degradation rate (η) can be expressed as:

(1) $$\eta =\left(C_{0}-C_{t}\right)/C_{0}\times 100\%$$

where η represents the degradation rate of dye; *C*_0_ represents the concentration of dye solution after dark reaction; and *C_t_* represents the concentration of dye solution at time *t*.

## 3. Results

### 3.1. Structure and Composition

**Figure 2:**
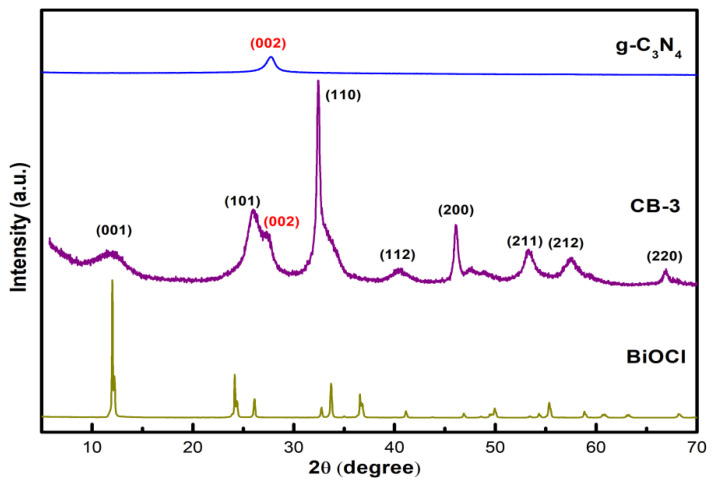
XRD patterns of g-C_3_N_4_, CB-3 and BiOCl.

Figure 2 shows the XRD patterns of the g-C_3_N_4_, BiOCl, and CB-3 heterojunction catalysts. BiOCl has characteristic diffraction peaks at 11.98°, 24.09°, 25.86°, 32.49°, 36.54°, 40.89°, 46.63°, 49.70°, 54.09°, 58.60°, and 68.03° which are assigned to the (001), (002), (101), (110), (003), (112), (200), (113), (211), (212), and (220) crystal planes, respectively. The results were consistent with the BiOCl standard spectrum (JCPDS06-0249). For g-C_3_N_4_, the strong diffraction peaks at 27.6° correspond to the (002) crystal planes of g-C_3_N_4_. For CB-3, the (001), (101), (110), (112), (200), (211), (212), and (220) crystal planes belonging to BiOCl can be observed, and the diffraction peak of the (110) plane is the strongest, which is consistent with the TEM results (BiOCl grows on carbon nitride along the (110) crystal plane). In contrast with those of the g-C_3_N_4_, the diffraction peak of the (002) crystal plane for CB-3 shifted slightly from 27.6° to 27.3°. The crystal plane spacing of the g-C_3_N_4_ increased according to the Bragg formula, owing to the BiOCl doped into the crystal planes of the g-C_3_N_4_.

**Figure 3:**
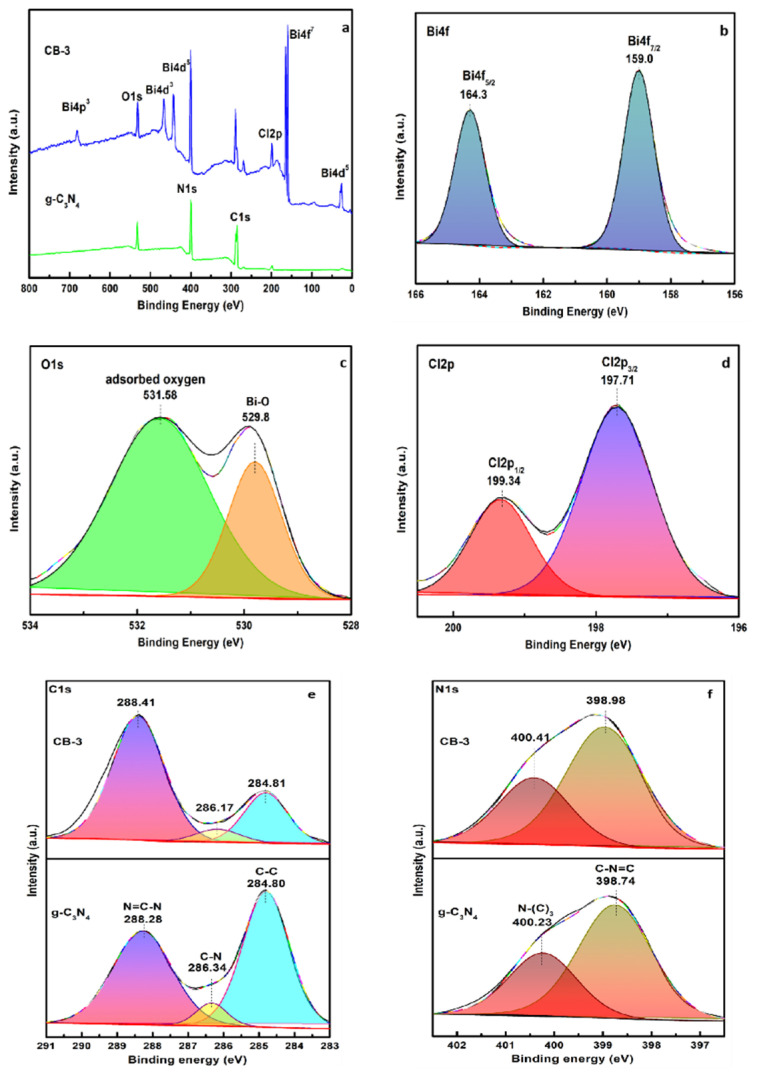
XPS spectra: (**a**) survey spectrum, (**b**) Bi4f, (**c**) O1s, (**d**) Cl2p, and (**e**) C1s, (**f**) N1s.

The chemical states of the g-C_3_N_4_ and CB-3 compositions were examined by XPS analysis (Figure 3). C, N, O, and Cl were found on the surface of the g-C_3_N_4_ (Figure 3a). The O was derived from the water adsorbed on the surface, and the Cl was derived from the cyanuric chloride doped into the g-C_3_N_4_. Furthermore, C, N, Cl, O, and Bi appear in the full spectrum of CB-3 (Figure 3a), and the peaks of N1s and C1s shifted to higher binding energies than those of the g-C_3_N_4_. The increase in binding energy reflects a decrease in electron cloud density, indicating that the C and N of the g-C_3_N_4_ interact with Cl and O, which are more electronegative, and that g-C_3_N_4_ and BiOCl recombined successfully. For g-C_3_N_4_, the high-resolution C1s spectrum can be fitted to three peaks (Figure 3e), which are related to the extraneous carbon (284.80 eV), C–N bonds (286.34 eV), and sp^2^ C–C bonds in the graphitic structure (288.28 eV). The N1s orbital can be fitted to two peaks with binding energies of 398.74 and 400.23 eV (Figure 3f), which are ascribed to sp^2^ hybridized N atoms in the triazine units (C=N–C) and tertiary nitrogen (N–(C)_3_), respectively [25,26,27,28]. For the C1s and N1s of the CB-3, the peaks obtained from the fitting are consistent with those of g-C_3_N_4_, and an increase in the binding energy can be observed (Figure 3e,f). Two different peaks can be observed in the high-resolution Bi4f spectra (Figure 3b). The two intense peaks at 159.0 and 164.3 eV are assigned to Bi 4f_7/2_ and Bi 4f_5/2_ [29], respectively. In Figure 3c, the two individual O1s peaks at 529.8 and 531.58 eV are ascribed to the lattice Bi-O and the H_2_O adsorbed on the surface, respectively. In Figure 3d, the two peaks at 197.71 and 199.34 eV are ascribed to Cl 2p_3/2_ and Cl 2p_1/2_, respectively.

### 3.2. Morphology

**Figure 4:**
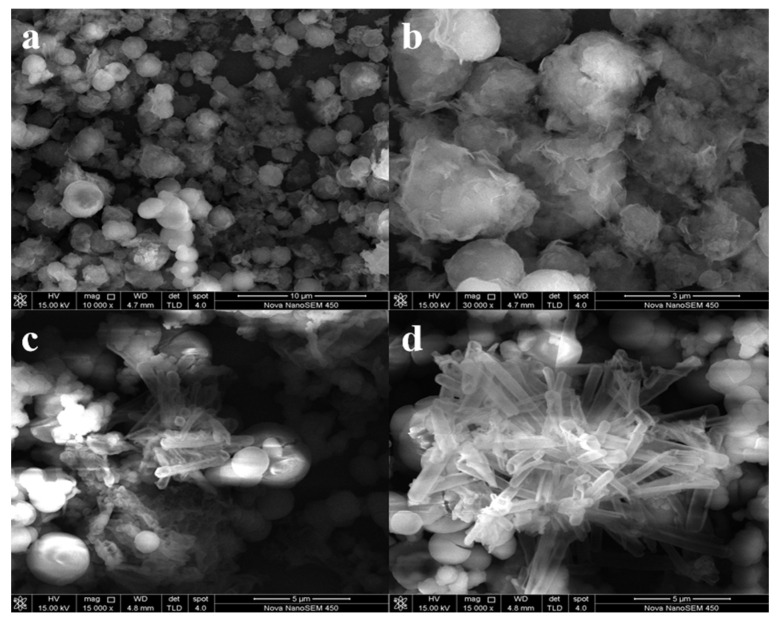
SEM of g-C_3_N_4_ and CB-3: (**a**,**b**) g-C_3_N_4_, (**c**,**d**) CB-3.

**Figure 5:**
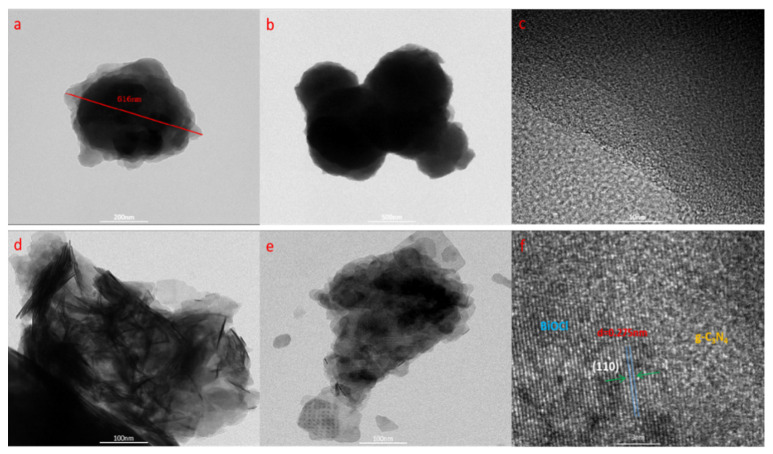
TEM of g-C_3_N_4_ and CB-3: (**a**–**c**) g-C_3_N_4_, (**d**–**f**) CB-3.

The morphology of g-C_3_N_4_ and CB-3 was investigated by SEM and TEM. The g-C_3_N_4_ prepared by the acetonitrile solvothermal method is mostly spherical (Figure 4a,b). The reasons for the formation of spherical g-C_3_N_4_ may be that cyanuric chloride is insoluble in acetonitrile, and the boiling point of acetonitrile is very low. In this homogeneous system, flaky g-C_3_N_4_ can self-assemble into spherical g-C_3_N_4_ [30]. The morphology of CB-3 is shown in Figure 4c,d and Figure 5d,e. The tubular BiOCl adheres densely to the spherical g-C_3_N_4_, demonstrating that g-C_3_N_4_/BiOCl composites were prepared successfully in one step, which is consistent with our interpretation of the XRD results. The TEM images of pure g-C_3_N_4_ (Figure 5a,b) show that the g-C_3_N_4_ remained spherical and has a diameter of 616 nm. However, flaky g-C_3_N_4_ was also observed, further demonstrating that the spheres are self-assembled from flaky g-C_3_N_4_. In contrast, the HRTEM results reveal an amorphous morphology [31] (Figure 5c). In addition, the HRTEM image of CB-3 shows that the (110) crystal planes of BiOCl appeared to border the amorphous g-C_3_N_4_ (Figure 5f), indicating that (110) crystal plane is the dominant exposed plane under acetonitrile solvothermal conditions.

**Figure 6:**
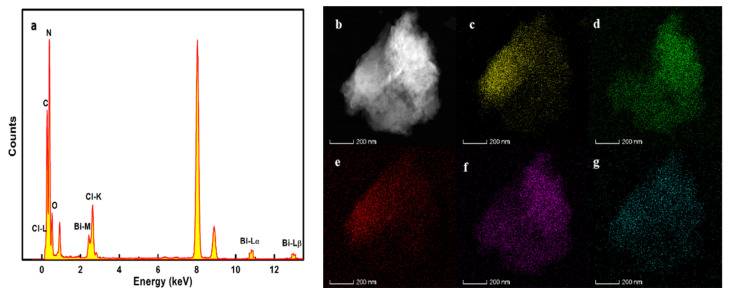
EDS and elemental mapping of CB-3: (**a**) EDS, (**b**) overlap, (**c**) C, (**d**) N, (**e**) Cl, (**f**) Bi, and (**g**) O.

EDS and elemental mapping best demonstrate the successful recombination of g-C_3_N_4_ with BiOCl. Figure 6a shows that the material is composed of C, N, O, Bi, and Cl, and the average atomic fraction of C/N/O/Bi/Cl is 44.64/39.20/7.13/5.65/3.38. The elemental mapping (Figure 6b–g) of CB-3 reveals that Bi, O, and Cl are well dispersed on the surface of g-C_3_N_4_, and Bi and O are somewhat clustered. These results prove that the heterojunction was successfully formed [32]. Otherwise, by comparing the atomic concentration determined by XPS with those obtained by EDS (Table 1), we think that the organic matter and oxygen supported on the surface of the catalyst cause the atomic fraction of the C and O to be slightly larger, respectively. The reason for the large atomic fraction of Cl is that the Cl of the raw material (cyanuric chloride) is doped into the catalyst. In addition, the weaker interaction of Bi and Cl may also lead to more point defects in BiOCl [33], thus leading to an error in atomic fractions. Furthermore, it is worth mentioning that the presence of point defects can be used as the active site of the reaction, which may be an important reason for enhancing photocatalytic activity.

### 3.3. Optical Properties

**Figure 7:**
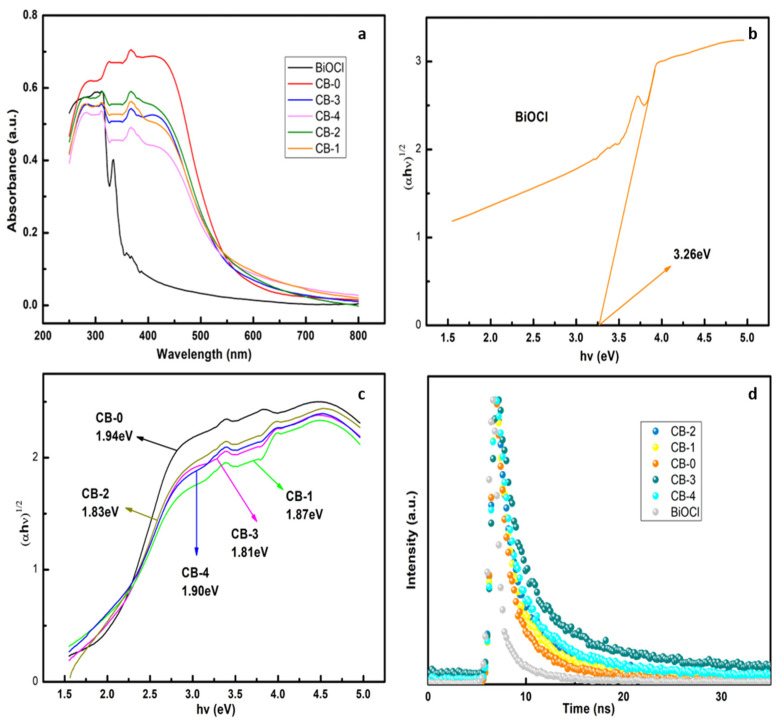
(**a**) UV-vis DRS, (**b**,**c**) Tauc plots, and (**d**) time-resolved fluorescence.

The optical properties of the samples were characterized by UV-vis spectra (Figure 7a). The band gaps of the CB-X composites and BiOCl were estimated using the Tauc formula:

(2) $$\alpha h\nu =A{\left(h\nu -E_{g}\right)}^{n/2}$$

where *α* is the absorption coefficient, *h* is Planck’s constant, *ν* is the frequency of light, and *A* is the proportionality constant. For indirect semiconductors, such as g-C_3_N_4_ and BiOCl, *n* = 4 [34], and *E_g_* is the sample band gap. BiOCl has a band gap of approximately 3.26 eV in Figure 7b. The absorption edge is approximately 380 nm, and it absorbs in the ultraviolet range. The band gap of g-C_3_N_4_ prepared by calcination is approximately 2.7 eV (the absorbing edge is approximately 460 nm) [35]. As shown in Figure 7c, the bandgap energy of the g-C_3_N_4_ prepared by the solvothermal method is approximately 1.94 eV, and the absorbing edge is expanded to 640 nm. Meanwhile, the band gaps of CB-X (X = 1, 2, 3, and 4) are 1.87, 1.83, 1.81, and 1.90 eV, respectively, and their absorption in the visible light region is better than that of g-C_3_N_4_ and BiOCl. The time-resolved fluorescence spectra of semiconductors can be used to study the lifetime, separation, and recombination of photogenerated electron-hole pairs [36]. The fluorescence lifetime of the material can be measured by the transient fluorescence spectra, and the average fluorescence lifetime of the material was calculated using the following formula:

(3) $$\tau =\left(\beta_{1}\tau_{1}^{2}+\beta_{2}\tau_{2}^{2}\right)/\left(\beta_{1}\tau_{1}+\beta_{1}\tau_{1}\right)$$

Long average fluorescence lifetimes equate to long electron-hole lifetimes, and the electrons and holes are well separated. Figure 7d shows the time-resolved fluorescence spectra of CB-X (X = 0, 1, 2, 3, and 4) and BiOCl at an excitation wavelength of 486 nm. The average fluorescence lifetimes of pure BiOCl and CB-0 are 2.48 and 6.04 ns, respectively. However, the average fluorescence lifetime of the composite material CB-3 is as high as 8.52 ns, which indicates that the electrons and holes in CB-3 are better separated. These results indicate that heterojunctions suppress photogenerated electron-hole recombination successfully.

### 3.4. Photocatalytic Activity

**Figure 8:**
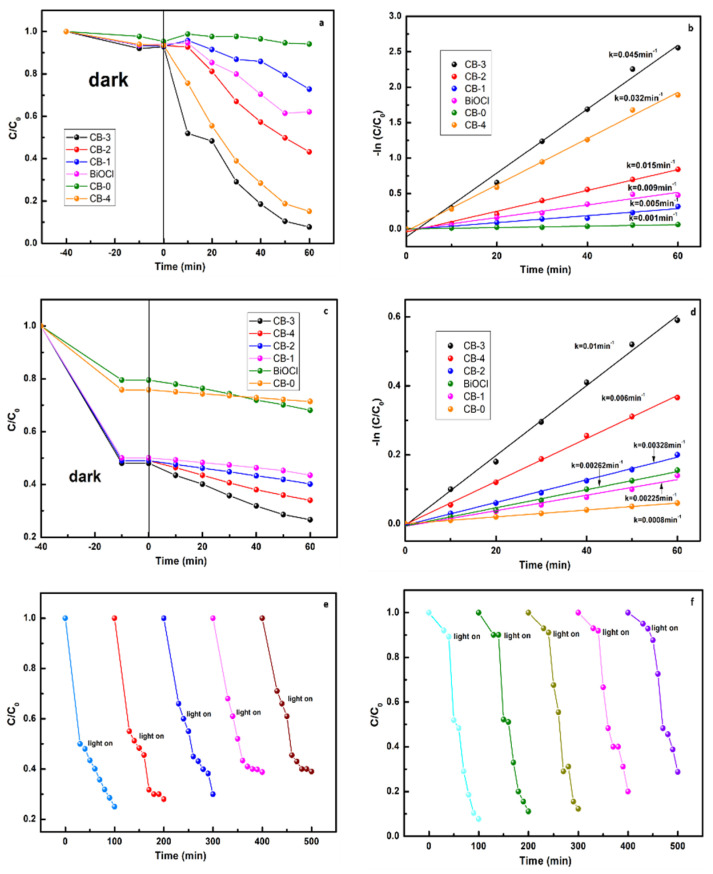
(**a**) Photodegradation efficiency of RhB, (**b**) pseudo-first-order kinetic model, (**c**) photodegradation efficiency of MO, (**d**) pseudo-first-order kinetic model, (**e**) reusability test of CB-3 for the photodegradation of MO, and (**f**) reusability test of CB-3 for the photodegradation of RhB.

The photocatalytic activity was measured by determining the efficiency of degrading RhB and MO (Figure 8a–d). After irradiation with visible light for 60 min, the degradation rate of RhB by CB-3 reached 93% and the degradation rates of RhB by CB-0 (g-C_3_N_4_) and BiOCl under the same conditions were 10% and 38%, respectively. The photocatalytic activity of the material significantly improved after the g-C_3_N_4_ was doped with BiOCl. To compare the photocatalytic performance of these catalysts, the kinetics of photodegradation were evaluated by applying the pseudo-first-order model:

(4) $$-\ln\left(C_{t}/C_{0}\right)=kt$$

where the *k* is the pseudo first-order rate constant, which represents the level of photocatalytic activity of the catalyst. The calculated rate constant of CB-3 is 0.045 min*^−^*^1^ (Figure 8b), and the rate constants of CB-0 and BiOCl are 0.001 min*^−^*^1^ and 0.009 min*^−^*^1^, respectively. The photodegradation rate of CB-3 is approximately 45 times higher than that of g-C_3_N_4_, indicating that the photocatalytic performance of the composite is very good, which means that the modification of one-step prepared g-C_3_N_4_ was very successful. In order to prove that the catalyst has a wide range of applicability, we additionally selected MO for degradation, and the method of processing the data is the same as that described above. After irradiation with visible light for 60 min, the degradation rate of MO by CB-3 reached 75% and the degradation rates of MO by CB-0 and BiOCl under the same conditions were only 20% and 30%, respectively. Otherwise, the apparent degradation rate constant attains a maximum value of 0.01 min^−1^, which is 12 times higher than that of g-C_3_N_4_ (less than 0.0008 min^−1^). The good stability and recyclability of the catalyst are necessary for photocatalytic reactions. As shown in Figure 8c, after three cycles of photodegradation of RhB and MO, the catalyst did not exhibit significant loss in activity. When the cycle of photodegradation reaches 5 times, the photocatalytic effect of the catalyst is reduced, but we believe that these losses are acceptable considering the loss in the catalyst and the adsorption of contaminants. Suitable control experiments without the addition of photocatalysts was conducted to determine the adsorption equilibrium of the photocatalysts and the effect of photolysis. The control experiments confirmed that the photocatalyst has physical adsorption on organic dyes in the dark reaction stage, and also the irradiation itself has no photolysis effect on the dyes.

To show the advantage of g-C_3_N_4_ nanoball/BiOCl nanotube heterojunction, the obtained results in this study have been compared with some reported catalysts in the literature [9,24,37,38,39,40], as summarized in Table 2. The most reported methods clearly require long reaction time or high concentration of catalyst. The nanocomposites synthesized in this study can achieve high removal ratio at short reaction time, so the present method is more suitable and superior.

### 3.5. Photoelectric Properties

**Figure 9:**
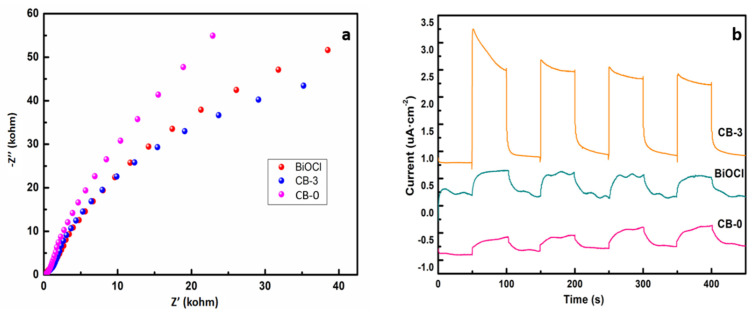
(**a**) Transient photocurrent responses, (**b**) electrochemical impedance spectroscopy.

The interfacial charge transport process directly reflects the carrier transport capability of photocatalytic materials to active sites [41]. The electrochemical properties of g-C_3_N_4_, BiOCl and CB-3 were investigated by electrochemical impedance spectroscopy and transient photocurrent. As shown in Figure 9a, the impedance of CB-3 is significantly lower than that of BiOCl and g-C_3_N_4_, indicating that the charge transfer resistance is low, which is beneficial to the improvement of photocatalytic performance. The transient photocurrent of CB-3 is larger (Figure 9b), which indicates that there are more surface electrons and holes, and that it exhibits the best photoelectric performance, which is consistent with the photocatalytic degradation results.

### 3.6. Photocatalytic Mechanism

**Figure 10:**
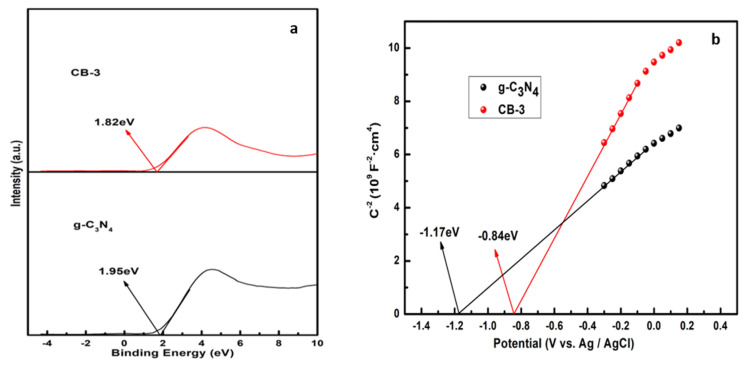
(**a**) VB-XPS, (**b**) Mott-Schottky plots.

The energy band position of the semiconductors is an important thermodynamic consideration for the photocatalytic activity. The VB-XPS in Figure 10a reveals the energy difference between the maximum VB and E_F_ [42]. The values for CB-3 and g-C_3_N_4_ are 1.82 and 1.95 eV, respectively. In Figure 10b, the Mott–Schottky plots reveal that the E_F_ values [43] for CB-3 and g-C_3_N_4_ are −0.84 and −1.17 eV (vs. Ag/AgCl), which are equal to −0.64 and −0.97 eV (vs. NHE), respectively. Thus, the VB values for CB-3 and g-C_3_N_4_ are 1.18 and 0.98 eV, respectively, and the corresponding CB values can be calculated by the formula:

(5) $$E_{CB}=E_{VB}-E_{g}$$

The *E_g_* values for CB-3 and g-C_3_N_4_ are 1.81 and 1.94 eV, respectively, according to the UV-Vis spectrum. As a result, the CB values for CB-3 and g-C_3_N_4_ are −0.63 and −0.96 eV, respectively. The results indicate that the valence band of the CB-3 increased, and the higher the VB top, the stronger the oxidation ability [44,45,46,47], which means that CB-3 exhibits better degradation activity.

**Figure 11:**
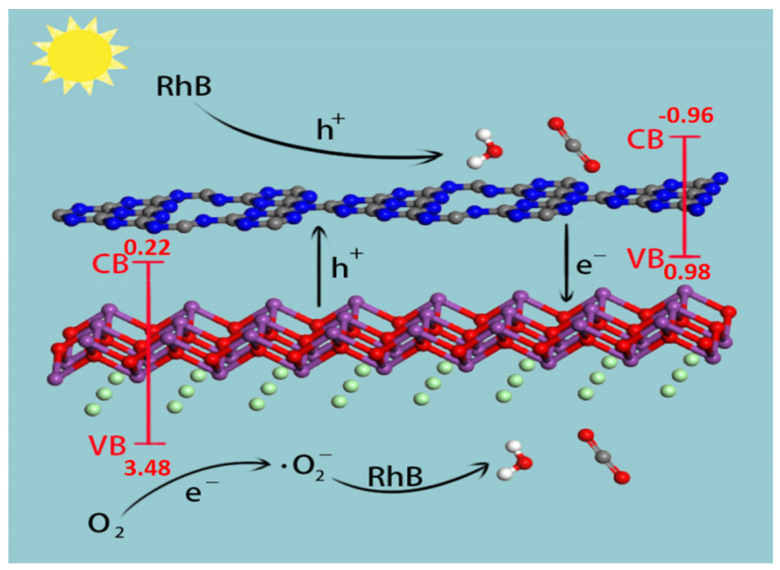
Plausible mechanism for the degradation of RhB.

The plausible charge carrier transfer mechanism of the step scheme heterojunction is depicted in Figure 11. The *E_CB_* and *E_VB_* of the BiOCl are 0.22 and 3.48 eV, respectively [48]. The BiOCl *E_VB_* potential (3.48 eV) is likely to be more positive than g-C_3_N_4_ (0.98 eV), whereas the *E_CB_* of g-C_3_N_4_ (−0.96 eV) is likely to be more negative than BiOCl (0.22 eV). As a result, the electrons and holes are successfully separated [49,50,51]. The former is transferred to the CB of BiOCl, and the latter are transferred to the VB of the g-C_3_N_4_, which improves the photocatalytic performance.

## 4. Conclusions

A g-C_3_N_4_/BiOCl composite material comprising a mixture of spheres and tubes was prepared by a one-step solvothermal method for the first time. The spherical g-C_3_N_4_ post BiOCl compounding demonstrated better photocatalytic activity than pure g-C_3_N_4_, and the quasi-primary reaction rate constant of RhB degradation was 45 times that of g-C_3_N_4_. Moreover, it demonstrated reasonable stability after five cycles, which proves that our low-temperature solvothermal method can successfully modify and optimize g-C_3_N_4_. Meanwhile, the new and special morphologies, using the low-temperature solvothermal method and the narrower band gap of catalysts, are desirable features that are likely to inspire further development. In summary, this research offers a feasible strategy to prepare diverse-shapes and high-performance catalysts for environmental protection and energy production.

## Figures and Tables

**Table 1:** Atomic concentration determined by XPS and EDS.

	C (%)	N (%)	O (%)	Cl (%)	Bi (%)
EDS1	51.99	31.57	6.64	5.94	3.86
EDS2	39.48	41.77	8.21	5.62	4.92
EDS3	42.44	44.27	6.55	5.38	1.36
AVERAGE	44.64	39.20	7.13	5.65	3.38
XPS	39.83	36.88	10.76	7.73	4.80

**Table 2:** Comparison of catalytic activity of g-C_3_N_4_ nanoball/BiOCl nanotube heterojunction with some reported catalysts in the degradation of organic dyes.

Catalyst	Dyes	Removal Ratio (%)	Time (min)	Dye (mg/L)	Catalyst (g/L)	Refs
g-C_3_N_4_/CdS/BiOCl	RhB	~90	30	20	1	[9]
g-C_3_N_4_/BiOCl	RhB	~90	150	20	1	[24]
g-C_3_N_4_/Bi_2_O_3_	RhB	~90	210	10	0.25	[37]
g-C_3_N_4_/BiOCl	RhB	~90	50	10	0.46	In this study
Ag/g-C_3_N_4_	MO	~95	300	20	2	[38]
BiOCl/Bi_12_O_17_Cl_2_	MO	~70	300	10	0.6	[39]
Cu/g-C_3_N_4_	MO	~90	70	10	0.5	[40]
g-C_3_N_4_/BiOCl	MO	~75	60	10	0.46	In this study

## Data Availability

The data presented in this study are available on request from the corresponding author.
